# Chronic renal insufficiency among Asian Indians with type 2 diabetes: I. Role of RAAS gene polymorphisms

**DOI:** 10.1186/1471-2350-7-42

**Published:** 2006-05-03

**Authors:** Pushplata Prasad, Arun K Tiwari, KM Prasanna Kumar, AC Ammini, Arvind Gupta, Rajeev Gupta, AK Sharma, AR Rao, R Nagendra, T Satish Chandra, SC Tiwari, Priyanka Rastogi, B Lal Gupta, BK Thelma

**Affiliations:** 1 Department of Genetics, University of Delhi South Campus, New Delhi, India; 2 Department of Endocrinology and Metabolism, M.S. Ramiah Medical College, Bangalore, India; 3 Department of Endocrinology, All India Institute of Medical Sciences, New Delhi, India; 4 Department of Nephrology, All India Institute of Medical Sciences, New Delhi, India; 5 Jaipur Diabetes and Research Centre, Jaipur, India; 6 Monilek Hospital and Research Centre, Jaipur, India; 7 Biometrics Division, Indian Agricultural Statistics Research Institute, New Delhi, India

## Abstract

**Background:**

Renal failure in diabetes is mediated by multiple pathways. Experimental and clinical evidences suggest that renin-angiotensin-aldosterone system (RAAS) has a crucial role in diabetic kidney disease. A relationship between the RAAS genotypes and chronic renal insufficiency (CRI) among type 2 diabetes subjects has therefore been speculated. We investigated the contribution of selected RAAS gene polymorphisms to CRI among type 2 diabetic Asian Indian subjects.

**Methods:**

Twelve single nucleotide polymorphisms (SNPs) from six genes namely-renin (REN), angiotensinogen (ATG), angiotensin converting enzyme I (ACE), angiotensin II type 1 receptor (AT1) and aldosterone synthase (CYP11B2) gene from the RAAS pathway and one from chymase pathway were genotyped using polymerase chain reaction-restriction fragment length polymorphism (PCR-RFLP) method and tested for their association with diabetic CRI using a case-control approach. Successive cases presenting to study centres with type 2 diabetes of ≥2 years duration and moderate CRI diagnosed by serum creatinine ≥3 mg/dl after exclusion of non-diabetic causes of CRI (n = 196) were compared with diabetes subjects with no evidence of renal disease (n = 225). Logistic regression analysis was carried out to correlate various clinical parameters with genotypes, and to study pair wise interactions between SNPs of different genes.

**Results:**

Of the 12 SNPs genotyped, Glu53Stop in AGT and A>T (-777) in AT1 genes, were monomorphic and not included for further analysis. We observed a highly significant association of Met235Thr SNP in angiotensinogen gene with CRI (O.R. 2.68, 95%CI: 2.01–3.57 for Thr allele, O.R. 2.94, 95%CI: 1.88–4.59 for Thr/Thr genotype and O.R. 2.68, 95%CI: 1.97–3.64 for ACC haplotype). A significant allelic and genotypic association of T>C (-344) SNP in aldosterone synthase gene (O.R. 1.57, 95%CI: 1.16–2.14 and O.R. 1.81, 95%CI: 1.21–2.71 respectively), and genotypic association of GA genotype of G>A (-1903) in chymase gene (O.R. 2.06, 95%CI: 1.34–3.17) were also observed.

**Conclusion:**

SNPs Met235Thr in angiotensinogen, T>C (-344) in aldosterone synthase, and G>A (-1903) in chymase genes are significantly associated with diabetic chronic renal insufficiency in Indian patients and warrant replication in larger sample sets. Use of such markers for prediction of susceptibility to diabetes specific renal disease in the ethnically Indian population appears promising.

## Background

Chronic renal insufficiency (CRI) due to type 2 diabetes is the leading cause of end-stage renal disease (ESRD) worldwide. This is one of the most important causes of premature death among patients with diabetes and a major health concern. Development of progressive renal disease is observed in only a proportion (20–30%) of individuals with type 2 diabetes [1] and familial clustering (2–4) provide clear evidence for genetic contribution though causative molecular mechanism(s) are unclear. High prevalence of diabetic kidney disease without a clear mode of inheritance even in familial cases cannot be explained on the basis of polymorphism/mutation in single gene but implies a multifactorial origin. Identification of such genetic determinants would be essential for a possible early intervention to prevent ESRD.

Candidate genes from various biochemical pathways such as aldose reductase-polyol, di-acyl glycerol-protein kinase C, advanced glycosylation-end products (AGE), hexosamine pathway and renin-angiotensin-aldosterone system (RAAS) have been implicated in the development of diabetic kidney disease. Of these, the involvement of RAAS gene polymorphisms in the pathogenesis of diabetic kidney disease has been abundantly studied in various populations [5-9]. The effector peptide of the RAAS, angiotensin II (Ang II), is a potent vasoconstrictor and the increase in intraglomerular pressure caused by Ang II results in proteinuria and glomerulosclerosis. Thus, all the genes or genetic loci responsible for excess Ang II production or availability are potential candidates for development of diabetic kidney disease. These include all the components of RAAS namely, renin (REN), angiotensinogen (AGT), angiotensin-1 converting enzyme (ACE), angiotensin II type1 receptor (AT1R) and aldosterone synthase (CYP11B2). In addition, chymase (CMA), a non RAAS gene with ACE like activity, also regulates physiological levels of Ang II. The physiological role of products of each of the above-mentioned genes in kidney function is well understood. Of all the markers tested for association with diabetic kidney disease, ins/del polymorphisms in ACE and Met235Thr SNP in AGT genes are the two most extensively studied. These polymorphisms are known to control the circulatory and cellular levels of their respective gene products [10-12], which in turn could control the expression of Ang II, providing a link between constitutive activity of RAAS and development of diabetic kidney disease.

Asian Indians, an ethnically distinct population, lead the world in the number of people with type 2 diabetes [13] and a consequent epidemic of diabetes associated micro- and macro-vascular complications is inevitable in India. Though reports on familial clustering of diabetic retinopathy and nephropathy [14,15], and a few candidate gene association studies in type 2 diabetes and diabetic retinopathy among Asian Indians are now available [16-18], very little is known about the susceptibility to diabetic renal disease, necessitating the present investigation. Considering the importance of the six genes of RAAS pathway in the development of diabetic glomerulopathy, association of several single nucleotide polymorphisms (SNPs)/SNP haplotypes in these genes that have been previously investigated in the other populations have been analysed with CRI in Indian subjects with type 2 diabetes in the present study. This is one of the first comprehensive reports on RAAS pathway gene polymorphisms and diabetic renal disease from the Asian Indian population.

## Methods

Subjects for the study were recruited at the four participating medical institutions situated across the country, namely MS Ramiah Medical College (Bangalore), All India Institute of Medical Sciences (New Delhi), Jaipur Diabetes and Research Centre (Jaipur), and Monilek Hospital and Research Centre (Jaipur). Ethical committee clearance was obtained from the respective medical institutions prior to the recruitment of subjects in this study. An informed consent was obtained from all the participants prior to their recruitment for the study.

### Subjects

Consecutive subjects suffering from Type 2 diabetes with CRI (cases, CRI, n = 196) and diabetics without any evidence of diabetic kidney disease (controls, DM, n = 225) were recruited from the out-patient departments at the study centres. Clinical data included information on duration of diabetes, presence of any complication, history of other disorders, weight (kg), height (cm), BMI (kg/m^2^), systolic and diastolic blood pressure. Documentation of antidiabetic and antihypertensive medication could not be obtained for the large majority of patients and hence these data are not reported. All subjects receiving treatment or those with blood pressure ≥140/≥90 mm Hg were considered hypertensive in the present study. All patients underwent either a fundoscopic examination or fluoroangiographic study for diagnosis of retinopathy. 10 ml venous blood was collected from each individual included in the study for biochemical and genetic analysis. Biochemical analyses to determine fasting glucose, glycated haemoglobin, serum creatinine, triglycerides, total cholesterol, and albumin were carried out at the respective centres using automated analyzer (Konelab 20, Thermoclinical lab systems, OY Finland) and similar protocols to ensure standardization. Glomerular filtration rate (GFR) was estimated by using online Cockcroft-Gault calculator [19]. For CRI, the inclusion criteria were subjects with Type 2 diabetes for ≥2 years, serum creatinine ≥3 mg/dl, urinary albumin excretion rate (AER) > 200 mg/l and presence of diabetic retinopathy. Patients with drug induced nephrotoxic damage or secondary causes of albuminuria such as obstructive renal disease, renal stone disease and acute urinary tract infection were excluded from this group. Normoalbuminuric (AER < 20 mg/l) Type 2 diabetes subjects of ≥10 years duration of diabetes (average 17.07 ± 6.69 years) were recruited as control diabetes subjects. Normoalbuminuria among control (DM) subjects was determined by timed urine collections. An aliquot of blood from the four centres was transported to the genetic laboratory (BKT) for DNA isolation. DNA was isolated from the lymphocytes using the conventional phenol-chloroform organic extraction method [20] and used for genetic analysis.

### Genetic analysis

Twelve polymorphisms from six genes namely REN, AGT, ACE, CMA, AT1 and CYP11B2, were chosen for genotyping using the polymerase chain reaction-restriction fragment length polymorphism (PCR-RFLP) approach, details of which are presented in Table 1. The choice of these SNPs was based on either their functional status or their widely analysed status. Primers were designed using Primer 3 software [21]. The digested PCR products were resolved on 2–3% agarose gel stained with ethidium bromide. Genotype configurations of each of the SNPs are also presented in this table.

**Table 1:** SNPs in RAAS pathway genes, their location, primer sequences, PCR conditions and restriction enzyme with product sizes.

**Polymorphism**	**Primer sequence**	**Product size (bp)**	**Annealing temp./Restriction enzyme/allele sizes**
*REN C>T (-4063) Promoter	F: 5'-AAA CTA GAA TGG GCT ACC AGA-3' R: 5'-GCT GTG ACT TGT CTC TTC CTG A-3'	235	60°C/Taq I/ C = 163, 68 T = 235
*REN (ACAG)n Intron G	F: 5'-ACA GTA CCTT CCC TCC TC TAC TCA-3' R: 5'-CTC TAT GGA GCT GGT AGA ACC TGA-3'	255	^a^1 = 255 2 = 259 3 = 263 4 = 267 5 = 271
*AGT A>G (-6) Promoter	F: 5'-CCC TCA GCT ATA AAT AGA GCA TC-3' R: 5'-GCA GGA AGA CCT GAC CAT CT-3'	308	61°C/Bst NI/ G = 245, 63 A = 36,209,63
*AGT Glu53Stop Exon 2	F: 5'-ACC ATC CTC TGC CTC CTG-3' R: 5'-TCC AAG GCT CCC AGA TAG AG-3'	398	58°C/PvuII/ C = 102, 102, 194 T = 204, 194
*AGT Thr174Met Exon 2	F: 5'-CAA TTC AGG CCA AGA CAT CC-3' R: 5'-GCC AGA GCC AGC AGA GAG-3'	674	58°C/Nco I/ C = 502, 172 T = 256, 246, 172
*AGT Met235Thr Exon 2	F: 5'-GAT GCG CAC AAG GTC CTG-3' R: 5'-CAG GGT GCT GTC CAC ACT GGC TCG C-3'	302	57°C/BstUI/ T = 302 C = 278, 24
ACE Ins/Del Intron 16	F: 5'-CTG GAG ACC ACT CCC ATC CTT TCT-3' R: 5'-GAT GTG GGC ATC ACA TTC GTC AGA T-3' **[Ref: Nucleic Acids Res. 1992, 20: 1433]**	490	Ins = 490 Del = 190
*AT1 A>C (-1166) Promoter	F: 5'-AAG AAG CCT GCA CCA TGT TT-3' R: 5'-CCA TCT TAC GGG CAT TGT TT-3'	626	58°C/Dde I/ A = 536, 90 C = 119, 417, 90
*AT1 A>T (-777) Promoter	F: 5'-AGT CAC CCT ACT CAC CTA GCT AAC A-3' R: 5'-AGA CAT CAC GAG ACT ACA GAT CAA-3'	192	60°C/Alu I/ A = 173 19 T = 192
*CYP11B2 T>C (-344) Promoter	5'-TGG AGG GTG TAC CTG TGT GTC A-3' 5' GTC CTG CTG GTC TGA GGA TG3'	270	57°C/Hae III/ T = 189, 81 C = 118, 71, 81
*CMA G>A (-1903) Promoter	F: 5'-GGA AAT GTGAGC AGA TAG TGC AGT C-3' R: 5'-AAT CCG GAGCTG GAG AAC TCT TGTC-3'	285	55°C/BstXI/ G = 195, 90 A = 285
*CMA C>T (-1777) Promoter	F: 5'-ATA TCA GTG AAA GCA AAC AGC TT-3' F: 5'-TAA GTG CCT TTC CCA AAT CA-3'	555	60°C/Mnl I/ C = 54, 501 T = 555

* Primers for these SNPs are designed in this study

** Alleles of the tetra nucleotide repeat (ACAG)n are resolved on PAGE and denoted as 1,2,3, 4 and 5.

### Statistical analysis

All the statistical tests were done using the SPSS version 11.0. Discrete and continuous variables were compared between cases (CRI) and controls (DM) using Pearson's χ^2 ^test and unpaired t-test as appropriate. Parameters with skewed distribution (diastolic and systolic blood pressure, and serum creatinine) are presented as median and range and compared using Mann-Whitney U test. Hardy-Weinberg equilibrium was tested for each of the SNPs based on the genotyping of 440 chromosomes from normal healthy individuals (average age 35.11 ± 8.98 years). These were recruited on a random basis from different locations including public meeting places, offices, colleges, markets and hospitals and represent population based controls. Pearson's χ^2 ^test (3 × 2 contingency table) was used to assess association of SNPs with renal disease using the cases (CRI) and controls (DM). Allelic and genotypic associations of SNPs found significant by Pearson's χ^2 ^test were evaluated by computing odds ratio (O.R.) and 95% confidence intervals (CI). Power of the sample size for each of the SNPs was calculated using PAWE software version 1.2 [22,23]. Haplotype analysis was performed using PHASE-standard analysis version 2.0.2 [24,25]. Chi-square values were derived from a series of 2 × 2 contingency tables based on the frequency of each haplotype versus all others between the diabetes (DM) and the CRI groups. Logistic regression analysis was carried out to correlate various clinical parameters with genotypes and to study pair wise interactions between SNPs of different genes. P values were subject to Bonferroni's correction and considered significant when <0.05.

## Results

### Clinical analysis

Demographic and clinical details of the subjects included in the study are given in Table 2. Total number of males as compared to females was more in both diabetes mellitus (DM) and CRI groups but male to female ratio between the two groups was not significantly different. No significant difference was observed in body-mass index, glycated hemoglobin (HbA1c), total serum cholesterol and triglyceride between the DM and CRI groups (P > 0.05). Serum creatinine, diastolic and systolic pressure, proportion of hypertensive individuals, and those with diabetic retinopathy were significantly higher (P < 0.05) among CRI subjects as compared to controls (Table 2).

**Table 2:** Clinical characteristics of the study population

**Characteristics**	**DM^a ^(N = 225)**	**CRI^b ^(N = 196)**	**P**
Gender (M/F)	76/149	65/131	0.89^d^
Age (years)	60.6 ± 11.5	57 ± 12.8	0.10^c^
Duration of Diabetes (years)	17.07 ± 6.69	10.4 ± 7.7	<0.05^c^
Hb A_1c_(%)	7.3 ± 1.0	7.5 ± 1.1	0.949^c^
Systolic pressure (mm Hg) *	140 (106–190)	150 (110–210)	0.002^e^
Diastolic pressure (mm Hg) *	84 (80–104)	90 (70–110)	0.025^e^
Serum creatinine (μmol/l) *	84 (35–108)	177 (124–1112)	<<0.05^e^
UAER (mg/l) *	10 (1–16)	864 (320–1584)	<<0.05^e^
GFR (mls/min/1.73 m^2^)	81.44 ± 21.85	27.83 ± 24	<<0.05^c^
Serum triglyceride (mmol/l)	1.7 ± 0.67	1.8 ± 1.4	0.307^c^
Serum cholesterol (mmol/l)	4.8 ± 0.97	4.9 ± 0.92	0.852^c^
Retinopathy (%) Non proliferative (%) Proliferative (%)	24 15 8.6	88 35 53	<<0.05^d^
Cardiovascular events (%)	3	8.3	

Data presented as mean ± SD (* median and range). ^a ^type 2 diabetes subjects without nephropathy (DM); ^b ^with diabetic renal insufficiency (CRI); ^c ^Student's t test; ^d ^Pearson's χ^2 ^test; ^e ^Mann-Whitney U test.

### Genetic analysis

Glu53Stop, a nonsense mutation in AGT gene and A>T (-777) SNP in AT1 gene were found to be monomorphic in our population and therefore not analysed further. Allele and genotype frequencies of the other ten SNPs along with their association status are presented in Table 3. We observed a highly significant association (P < 0.01) of Met235Thr SNP in AGT gene and odds ratio calculations identified Thr allele and Thr/Thr genotype to be predisposing to CRI (O.R. 2.68, 95%CI: 2.01–3.57 for Thr allele, O.R. 2.94, 95%CI: 1.88–4.59 for Thr/Thr genotype). A strong association of T allele (O.R. 1.57, 95%CI: 1.16–2.14) and TT genotype (O.R. 1.81, 95%CI: 1.21–2.71) of T>C -344 SNP of CYP11B2 gene and GA genotype (O.R. 2.06, 95%CI: 1.34–3.71) of G>A (-1903) promoter SNP of CMA gene was also observed in our sample set. A large number of haplotypes for AGT gene were generated and are presented in Table 4. Haplotype G-C-T (G-6 – Thr174 – Met235) was seen to be predominant among the total sample set (37% among DM and 31% among CRI) analysed in this study. Only two haplotypes A-C-C (A-6 – Thr174 – Thr235) and A-C-T (A-6 – Thr174 – Met235) were significantly associated (after multiple corrections) with CRI (χ^2 ^= 40.83, P < 0.01; χ^2 ^= 47.38, P < 0.01 respectively) and were found to be the predisposing and protective haplotypes respectively (Table 4).

**Table 3:** Allele and genotype frequencies of SNPs and their association status with diabetic chronic renal insufficiency.

**SNPs**	**Allele frequency**		**Genotype frequency**		**Association**	
	
	**DM**	**CRI**	**DM**	**CRI**	**Allele (df = 1)**	**Genotype (df = 2)**
REN C>T (-4063)	C = 0.85	C = 0.84	CC = 0.73	CC = 0.72	χ^2 ^= 1.01, P = 0.60	χ^2 ^= 0.29, P = 0.59
	T = 0.15	T = 0.16	CT = 0.24	CT = 0.23		
			TT = 0.03	TT = 0.05		
REN (ACAG)n	1 = 0.80	1 = 0.81	11 = 0.64	11 = 0.67	χ^2 ^= 8.56, P = 0.38 (df = 4)	χ^2 ^= 4.55, P = 0.34 (df = 8)
	2 = 0.10	2 = 0.105	12 = 0.155	12 = 0.14		
	3 = 0.09	3 = 0.07	13 = 0.155	13 = 0.10		
	4 = 0.01	4 = 0.016	14 = 0.02	14 = 0.026		
	5 = 0.00	5 = 0.005	22 = 0.01	22 = 0.02		
			23 = 0.02	23 = 0.02		
			33 = 0.00	24 = 0.005		
				33 = 0.01		
				55 = 0.005		
AGT A>G (-6)	G = 0.46	G = 0.41	GG = 0.32	GG = 0.24	χ^2 ^= 2.21, P = 0.14	χ^2 ^= 3.38, P = 0.18
	A = 0.54	A = 0.59	GA = 0.28	GA = 0.34		
			AA = 0.40	AA = 0.42		
AGT Thr174Met	C = 0.90	C = 0.87	CC = 0.81	CC = 0.76	χ^2 ^= 1.32, P = 0.25	χ^2 ^= 1.48, P = 0.48
	T = 0.10	T = 0.13	CT = 0.18	CT = 0.23		
			TT = 0.01	TT = 0.01		
AGT Met235Thr	T = 0.61	T = 0.37	TT = 0.42	TT = 0.16	χ^2 ^= 46.41, P << 0.01	χ^2 ^= 39.2, P << 0.01
	C = 0.39	C = 0.63	TC = 0.38	TC = 0.42		
			CC = 0.20	CC = 0.42		
ACE Ins/Del	Ins = 0.56 Del = 0.44	Ins = 0.53 Del = 0.47	Ins/Ins = 0.34 Ins/Del = 0.43 Del/Del = 0.23	Ins/Ins = 0.34 Ins/Del = 0.38 Del/Del = 0.28	χ^2 ^= 0.76, P = 0.38	χ^2 ^= 1.60, P = 0.45
AT1 A>C (-1166)	A = 0.93	A = 0.93	AA = 0.86	AA = 0.86	χ^2 ^= 0.08, P = 0.78	χ^2 ^= 0.20, P = 0.90
	C = 0.07	C = 0.07	AC = 0.13	AC = 0.11		
			CC = 0.01	CC = 0.01		
CYP11B2 T>C (-344)	T = 0.64	T = 0.73	TT = 0.40	TT = 0.54	χ^2 ^= 8.54, P = 0.003	χ^2 ^= 0.84, P = 0.01
	C = 0.36	C = 0.27	T C = 0.47	T C = 0.38		
			CC = 0.13	CC = 0.08		
CMA G>A (-1903)	G = 0.57	G = 0.52	GG = 0.27	GG = 0.13	χ^2 ^= 2.51, P = 0.11	χ^2 ^= 12.5, P = 0.002
	A = 0.43	A = 0.48	GA = 0.62	GA = 0.77		
			AA = 0.12	AA = 0.10		
CMA C>T (-1777)	C = 0.68	C = 0.71	CC = 0.49	CC = 0.50	χ^2 ^= 0.88, P = 0.35	χ^2 ^= 4.24, P = 0.12
	T = 0.32	T = 0.29	CT = 0.38	CT = 0.43		
			TT = 0.13	TT = 0.07		

**Table 4:** Haplotypes of A>G (-6) – Thr174Met – Met235Thr SNPs in AGT gene tested for association with diabetic chronic renal insufficiency.

Haplotype*	**DM**		**CRI**		**χ^2^**	**P (df = 2)**	**O.R. (95%CI)**
	**n**	**Freq.**	**n**	**Freq.**			
**A-C-T**	**92**	**0.236**	**23**	**0.06**	**47.38**	0.0000**	**0.260 (1.27–0.33)**
**A-C-C**	**93**	**0.24**	**174**	**0.45**	**16.99**	0.0000**	**1.93 (1.41–2.65)**
A-T-T	08	0.20	01	0.003	5.4	0.02	0.125 (0.015–1.00)
A-T-C	16	0.04	30	0.08	4.76	0.029	1.98 (1.06–3.69)
G-C-T	144	0.37	118	0.31	3.32	0.068	0.758 (0.56–1.02)
G-C-C	25	0.06	25	0.065	0.06	0.81	0.93 (0.52–1.67)
G-T-T	06	0.015	01	0.003	3.526	0.06	0.167 (0.02–1.39)
G-T-C	06	0.015	14	0.036	3.14	0.076	2.34 (0.89–6.15)

* Order of SNPs in AGT haplotypes: A>G (-6) – Thr174Met – Met235Thr

** Significant after Bonferroni correction α = 0.006

In a multiple logistic regression analysis (backward stepwise logistic regression method) using DM or CRI status as a dependent variable, we observed a significant association of Met235Thr polymorphism with CRI (P < 0.01) and Thr/Thr genotype (O.R. 1.54, 95%CI: 1.03–2.04) was found to be highly predisposing supporting the results obtained from Pearson's χ^2 ^test and odds ratio analysis. Pair-wise interactions between different polymorphisms included in this study were tested using multiple logistic regression analysis. No significant interaction between any of these polymorphisms/genes was observed.

## Discussion

Considering that India leads the world with the highest number of individuals affected with Type 2 diabetes very little information on the genetic susceptibility to this complex trait as well as to several of diabetes specific micro- and macro-vascular complications among Indian subjects is available. In this report on RAAS pathway genes, we tested association of a total of twelve polymorphisms from exonic, intronic and promoter regions of six genes with diabetic CRI using a case-control approach and found significant associations of RAAS and chymase pathway genes, viz., Met235Thr in angiotensinogen, T>C (-344) in aldosterone synthase, and G>A (-1903) in chymase genes.

There was no significant difference in either the gender distribution or age in the two groups included in our study. Duration of Type 2 diabetes was significantly different between the two groups, with control diabetes group having a longer duration, which ensured the choice of appropriate controls in our study. Significantly higher diastolic and systolic blood pressure, as well as higher percentage of diabetic retinopathy and cardiovascular disease were observed among CRI subjects in our study (Table 2), which is in conformity with common clinical observations in diabetic kidney disease [26-28].

There are a few study limitations. Firstly, the study involves a small sample size. Harrington has commented on standards of evidence in the genomic age and suggests that in a case control study the sample size should be >1000 subjects to arrive at worthwhile conclusions [29]. However, only a very few investigators have access to such a large data and inspite of being a multi-site study the present study could not achieve such large numbers. On the other hand the present study fulfils most of the criteria of a good genetic association study suggested by Hattersley et al [30]. Secondly, the diagnosis of diabetic renal disease in the present study is based on multiple criteria. Diabetes can produce a large variety of renal lesions and it is likely that molecular and genetic pathways involved in each of these are different. In the present study all the cases had moderate to severe CRI as indicated by creatinine of ≥3 mg/dl and involvement of various components of RAAS pathway is clearly indicated in advanced diabetic kidney disease by many previous studies. Thirdly, lack of treatment details of the subjects is a minor limitation as treatment with ACE inhibitors or ARBs do not influence genetic polymorphisms.

Of the ten polymorphisms that were genotyped in the study groups (Table 3), tetra nucleotide repeat (ACAG)_n _of REN gene and SNPs A>G (-6) and Met235Thr of AGT gene were not in Hardy-Weinberg-Equilibrium (HWE) in the present study (data not shown). Higher rate of mutation/variability of microsatellite marker(s) may provide a likely explanation for deviation of (ACAG)_n _of REN gene from HWE. The deviation of SNPs A>G (-6) and Met235Thr of AGT gene may be due to significant excess of heterozygotes of both the SNPs in our representative baseline control population, which could be attributed to yet unknown selection pressure. We confirm that care was taken to ensure random sampling of population based controls and also error free genotyping as reported in previous studies from our group [31]. We did not observe association of SNPs in three genes namely REN, ACE and AT1 with CRI in our study. The results in renin gene is not surprising since association of the two polymorphisms in REN gene included in this study has not been reported so far in any population. However, there are many reports on association in AT1 [32-34] and ACE genes [35-38], which are inconsistent across populations. The only other published study on ACE ins/del polymorphism in diabetic nephropathy subjects from India showed a significant association of the del allele [39].

Met235Thr in AGT gene showed a very strong allelic, genotypic and haplotypic association with CRI (Tables 3, 4). This is quite in agreement with data from functional analysis of this molecular variant, where in Thr/Thr homozygous individuals were shown to have elevated levels of angiotensinogen which could then lead to increased production of vasoconstrictor peptide Ang II in blood plasma and kidney [12]. Contrary to an earlier observation [12] we neither observed a tight LD between Met235Thr and the promoter SNP A>G (-6) of this gene nor association of the promoter SNP with CRI. Our observation of independent contribution of Met235Thr to development of CRI is further supported by our results of haplotype analysis. Out of the two significantly associated haplotypes, the haplotype A-C-C containing Thr235 is predisposing to CRI (χ^2 ^= 40.83; P << 0.01) and another haplotype A-C-T- (A-6 – Thr174 – Met235), with only one allele (Met235) different as compared to the predisposing haplotype, is protective (χ^2 ^= 47.38; P << 0.01) to CRI (Table 4). It may be added here that haplotype association observed in this study, is genuine and not caused by the deviation of this marker from HWE, since any haplotype analysis carried out using PHASE (Version 2.0.2) software is not influenced by HWE. Multiple logistic regression analysis results, which showed a very significant association of only Met235Thr SNP and Thr235 allele even in the presence of all other genetic and crucial clinical factors analysed in the study, further underscores the involvement of 235Thr allele. 41% of all CRI patients in our sample set possessed Thr/Thr genotype. Based on all these observations, and considering the power of our sample (G = 99%) we conclude that Thr235 could be a susceptibility allele for CRI. The role of Met235Thr polymorphism in the development of diabetic nephropathy and ESRD is widely debated and published association studies present inconsistent results [40-44].

SNP T>C-344 of aldosterone synthase gene was also found to be significantly associated (P = 0.012) with CRI in our sample set. Allele T and genotype TT of this SNPs seems to be predisposing to kidney disease (O.R. 1.57; 95%CI: 1.16–2.14 and O.R. 1.81; 95%CI: 1.21–2.71 respectively) but we would be cautious of this association due to the sub-optimal power of the sample (G = 40%). Recent experimental evidences implicate aldosterone as an important factor in pathogenesis of advanced diabetic kidney disease independent of arterial blood pressure and plasma Ang II levels [45]. In addition, it has been observed that "aldosterone escape" observed in a proportion of DN patients during blockade of RAAS is associated with decline in glomerular filtration rate [46].

We also observed a significant association of G>A (-1903) promoter SNP of chymase gene with CRI. No allelic association was observed but genotype GA seems to be highly predisposing (O.R. 2.06; 95%CI: 1.34–3.17) to diabetic renal disease. Chymase gene plays an important role in Ang II production under conditions like diabetes mellitus and glomerular hypertension. Considering such an importance of CMA gene in renal function our observation of association of this promoter SNP in this gene seems exciting but due to the low power of our sample (G = 14%) this result needs to be replicated in larger sample sets. However, the only other study on European population, did not observe association of this SNP with DN [47].

Pearson's χ^2 ^test carried out to test the possibility of association of the polymorphisms analysed in this study with hypertension (data not shown) showed no association. This further confirmed the independent association of Met235Thr, T>C (-344) and G>A (-1903) SNPs with CRI and suggests that these SNPs though known to modulate RAAS activity, does not operate through hypertension as a crucial mechanism but via stimulation of chemokines like TGF β1, TNF α and IL1 [48,49], thus warranting further investigations of such genes.

## Conclusion

Use of SNPs Met235Thr in angiotensinogen, T>C (-344) in aldosterone synthase, and G>A (-1903) in chymase genes for prediction of susceptibility to diabetes specific renal disease in the Asian Indian population appears promising. However, results from this pilot study not only warrant replication in larger sample sets obtained from multiple centres across the country but also investigations on candidate gene polymorphisms from other biochemical pathways.

## Competing interests

The author(s) declare that they have no competing interests.

## Authors' contributions

PP was involved in the study design, carried out molecular genetics and statistical analyses, compiled the data, wrote the Ms.; AKT was involved in study design and molecular genetic analysis; KMPK, ACA, AG, AKS and RG were the principal clinical investigators involved in study design, defining exclusion and inclusion criteria of study subjects and were mainly responsible for identification of study subjects from their respective clinical centres; ARR was involved in statistical analysis, RN and TSC were responsible for recruitment of identified subjects, collecting detailed clinical records and documentation of data and day to day follow up of the project at their respective centres, SCT was the consultant nephrologist and contributed to study design; TBK was the principal geneticist and co-ordinator of the project, involved in conceptualization of the project, study design, oversee complete genetic analyses in the laboratory, critical inputs and finalization of the manuscript.

## Pre-publication history

The pre-publication history for this paper can be accessed here:


